# Tauroursodeoxycholic acid as a beneficial modulator for developmentally programed chromatin structure around specific genes

**DOI:** 10.3389/fendo.2024.1211657

**Published:** 2024-08-26

**Authors:** Hiroaki Itoh, Tomoko Aoyama, Yukiko Kohmura-Kobayashi, Naoaki Tamura, Takahiro Nemoto

**Affiliations:** ^1^ Department of Obstetrics and Gynecology, Hamamatsu University School of Medicine, Hamamatsu, Japan; ^2^ National Institutes of Biomedical Innovation, Health and Nutrition, Osaka, Japan; ^3^ Department of Bioregulatory Science (Physiology), Nippon Medical School, Tokyo, Japan

**Keywords:** pregnancy, fetus, developmental origins of health and disease (DOHaD), secondary bile acid, histone modifications, epigenome, ER stress

## Introduction

The research reported in ‘The Fetal Origins of Metabolic Disorders Volume II’ is derived from the theory of Developmental Origins of Health and Disease (DOHaD) (1, 2), which connects health disorders with environmental disruptions during early life stage. The DOHaD theory was historically devised from the findings of cohort studies of the long-term health deterioration of certain groups such as those that were born as small neonates or born from mothers experiencing the famine in World War II (1, 2). The findings were supported by animal studies. Epigenetics has provided powerful research tools that allow for the exploration of DNA methylation, histone modifications, and non-coding RNAs for the purpose of searching how adverse exposure in early life results in epigenetic and gene expression changes that contribute to the risk of chronic disease later in life (3). Recently, tauroursodeoxycholic acid (TUDCA), a secondary bile acid, has been used as a therapeutic strategy to minimize adipose tissue dysfunction and metabolic alterations associated with obesity (4). This editorial introduces the hypothesis that TUDCA beneficially remodels the chromatin structure around the genes associated with the developmentally programed obesity-prone phenotype of adults.

## TUDCA improves obesity-associated disorders

TUDCA has been used for centuries in Chinese medicine and is approved by the Food and Drug Administration for treatment of primary biliary cholangitis. Furthermore, TUDCA has potential therapeutic benefits in various diseases including diabetes, obesity, and neurodegenerative diseases. TUDCA has cytoprotective activity by alleviation of endoplasmic reticulum (ER) stress as a chemical chaperon-stabilizing unfold protein response (5). Furthermore, TUDCA induces beneficial metabolic effects by activating farnesoid X receptor and G protein-coupled bile acid receptor (4). However, the exact association between its receptor-mediated pathways and its activity as a chemical chaperon remains to be elucidated.

There have been limited numbers of studies that described the favorable effect of TUDCA treatment on DOHaD-associated models. Yung et al. reported the advantageous effect of TUDCA treatment on pregnant women with gestational diabetes (6). Pasha et al. reported that TUDCA treatment improved fetal growth in aged rat dams (7). Our research group reported that TUDCA treatment improved hepatic steatosis (8, 9) and fat pad deposition (10) specifically in adult mice experienced undernourished (UN) *in utero*.

## TUDCA induces beneficial changes of chromatin structure around *Cidea* and *Cidec* genes in pups with UN *in utero*

**Figure 1:**
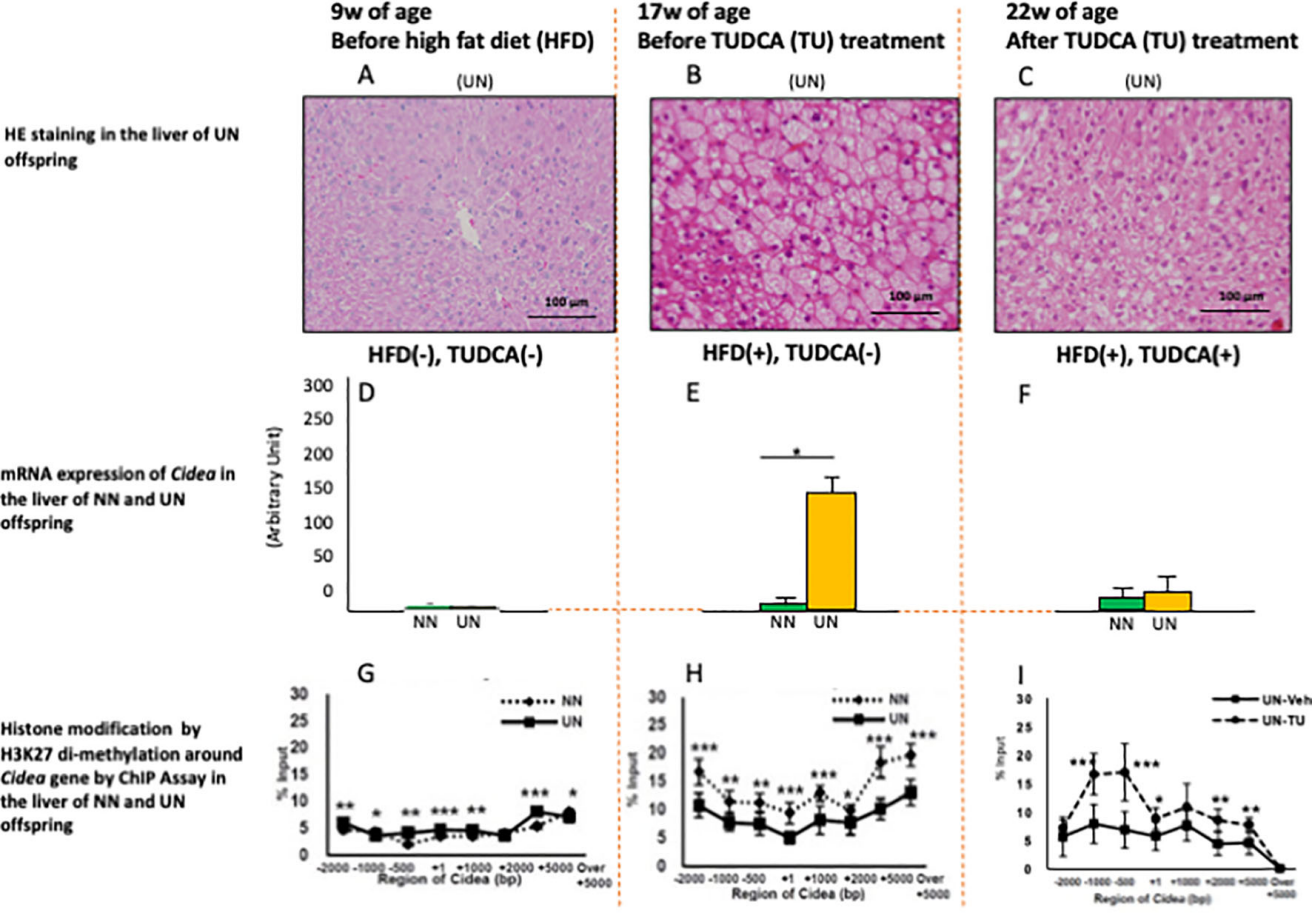
HE staining **(A–C)**, mRNA expression of *Cidea* gene **(D–F)**, and histone modification by H3K27 di-methylation around *Cidea* gene by ChIP Assay **(G–I)** in the liver of the mouse pups at 9w **(A, D, G)**, 17w **(B, E, H)** and 22w **(C, F, I)** of age with undernourishment (UN) or normal nourishment (NN) *in utero*, before or after high fat diet (HFD) and TUDCA (TU) treatment. *P < 0.05. **P < 0.01. ***P < 0.001. Cited from Urmi et al. (9).

Previous studies of cell models reported that TUDCA induces epigenetic changes (5); however, few studies have reported advantageous epigenetic effects in experimental DOHaD animal models. Urmi et al. reported that UN *in utero* caused deteriorated hepatic steatosis of adult pups under obesogenic diet (**Figures 1A, B**) concomitant with specific augmentation of *Cidea* gene expression (**Figures 1D, E**) and suppression of histone modification of H3K27 di-methylation (transcriptional promotion) around *Cidea* (**Figures 1G, H**) (9). Both *Cidea* and *Cidec* induce the fusion of lipid droplets and augment lipid deposition in hepatocytes. Moreover, TUDCA treatment improved hepatic steatosis in adult pups with UN *in utero* (**Figures 1B, C**), but not normally nourished pups (not shown). This occurred with specific inhibition of *Cidea* gene expression (**Figures 1E, F**) and upregulation of histone modification of H3K27 di-methylation (transcriptional repression) around *Cidea* (**Figures 1H, I**). Similar findings were also observed with *Cidec* (9). Urmi et al.’s findings suggest that histone remodeling by TUDCA improves hepatic steatosis in a DOHaD animal model; however, further studies using a gene deletion model are necessary to clarify if it is inevitable for the improvement of hepatic steatosis. Nevertheless, TUDCA treatment beneficially remodeled chromatin structures around some key genes of lipid deposition in adult pups with developmental programming by UN *in utero*, suggesting a possible promising future use of TUDCA as a beneficial modulator of developmentally programmed chromatin structure, which is applicable even in the adult period.

## Contributions of the articles published in the research topic

The research topics in ‘The Fetal Origins of Metabolic Disorders Volume II’ are: 1) environmental disruption in the early critical period, 2) resultant phenotypic disorders in offspring, and 3) the mechanism of programming. Regarding 1), Doi et al. (hyper link) reported the effects of methamphetamine exposure. Regarding 2), Deer et al. (hyper link) reviewed cardiovascular disease risk and Umeda et al. (hyper link) investigated fetal growth and polyunsaturated fatty acid metabolism. Regarding 3), Olive et al. (hyper link) investigated cortisol regulation and Gracia-Rizo et al. (hyper link) reported the association between glucose metabolism and first-episode psychosis.
